# Euglycemia in Diabetic Rats Leads to Reduced Liver Weight via Increased Autophagy and Apoptosis through Increased AMPK and Caspase-3 and Decreased mTOR Activities

**DOI:** 10.1155/2015/497431

**Published:** 2015-04-28

**Authors:** Jun-Ho Lee, Soo-Bong Choi, Mingli Jin, Ju-Han Lee, Sang-Don Han, Hyemi Bae, Inja Lim, Yun-Hee Noh

**Affiliations:** ^1^Department of Biochemistry, School of Medicine, Konkuk University, 120 Neungdong-ro, Gwangjin-gu, Seoul 143-701, Republic of Korea; ^2^Department of Internal Medicine, School of Medicine, Konkuk University, Chungju Hospital, 82 Kukwondae-ro, Chungju 380-704, Republic of Korea; ^3^Rmedica-Stem Cell, 98 Gasan Digital 2-ro, Geumcheon-gu, Seoul 153-768, Republic of Korea; ^4^Department of Neurology, School of Medicine, Konkuk University, Chungju Hospital, 82 Kukwondae-ro, Chungju 380-704, Republic of Korea; ^5^Department of Physiology, College of Medicine, Chung-Ang University, 84 Heukseouk-ro, Dongjak-gu, Seoul 156-861, Republic of Korea

## Abstract

Euglycemia is the ultimate goal in diabetes care to prevent complications. However, the benefits of euglycemia in type 2 diabetes are controversial because near-euglycemic subjects show higher mortality than moderately hyperglycemic subjects. We previously reported that euglycemic-diabetic rats on calorie-control lose a critical liver weight (LW) compared with hyperglycemic rats. Here, we elucidated the molecular mechanisms underlying the loss of LW in euglycemic-diabetic rats and identified a potential risk in achieving euglycemia by calorie-control. Sprague-Dawley diabetic rats generated by subtotal-pancreatectomy were fed a calorie-controlled diet for 7 weeks to achieve euglycemia using 19 kcal% (19R) or 6 kcal% (6R) protein-containing chow or fed* ad libitum* (19AL). The diet in both R groups was isocaloric/kg body weight to the sham-operated group (19S). Compared with 19S and hyperglycemic 19AL, both euglycemic R groups showed lower LWs, increased autophagy, and increased AMPK and caspase-3 and decreased mTOR activities. Though degree of insulin deficiency was similar among the diabetic rats, Akt activity was lower, and PTEN activity was higher in both R groups than in 19AL whose signaling patterns were similar to 19S. In conclusion, euglycemia achieved by calorie-control is deleterious in insulin deficiency due to increased autophagy and apoptosis in the liver via AMPK and caspase-3 activation.

## 1. Introduction

Type 2 diabetes (T2D) is a progressive metabolic disease characterized by hyperglycemia due to a combination of insulin resistance and defective insulin secretion [1, 2]. The extent of hyperglycemia is associated with the development of diabetic complications. Thus, correction of hyperglycemia to euglycemia, or near-euglycemia, has been the ultimate goal [3–5]. However, because intensive glycemic control increases both cardiovascular and overall mortality in T2D [6, 7], the latest guidelines for the management of hyperglycemia in T2D recommend a more flexible glycemic target taking into consideration an individual's clinical characteristics [8]. This recommendation creates a dilemma in that achieving euglycemia to prevent microvascular complications in diabetic patients [1, 4, 9–11] should then be reserved to reduce mortality in T2D, while the latest guidelines may let the patients with T2D survive longer but live with the disabilities caused by increased microvascular complications due to a relaxed glycemic target. Overcoming this dilemma requires a better understanding of how euglycemia differentially affects major organs/tissues according to the accompanying insulin and/or insulin signal level. This, in turn, may explain why euglycemia that prevents microvascular complications ultimately increases cardiovascular and overall mortality in T2D.

We recently reported that euglycemia achieved by diet control in diabetic rats (fed isocalorie per body weight compared with sham-control nondiabetic rats) is different from that in sham-control rats; the mean liver weight in the euglycemic-diabetic rats is significantly lower than in the sham-control rats due to increased autophagy [12]. In contrast, the hyperglycemic-diabetic rats on* ad libitum* diet maintained the same liver weight as the sham-control rats in the same study. Therefore, we previously suggested that hyperglycemia in the presence of insulin deficiency might protect hepatocytes against excessive autophagy. However, we have not investigated the protection mechanism against excessive autophagy in the livers of the hyperglycemic-diabetic rats and the mechanism for the excessive autophagy in the livers of the euglycemic-diabetic rats at the molecular level. Understanding these mechanisms may clarify whether intensive glycemic control to achieve euglycemia is more harmful than poor glycemic control in terms of the survival of hepatocytes when insulin deficiency exists, which is common in T2D, regardless of the degree of insulin resistance [13]. Importantly, chronic liver disease and/or hepatocellular carcinoma are the fourth most common causes of death among patients with T2D [14], so we speculated that when there is a preexisting liver ailment, euglycemia in the presence of insulin deficiency might increase liver damage, possibly leading to hepatic failure.

Autophagy is induced when cellular nutrient levels decrease, a process in which AMPK, a major energy sensor in most cells, is activated and mediates the autophagic process [15]. Conversely, when cellular nutrient levels increase, mTOR, a signaling molecule for protein synthesis, is activated and inhibits autophagy [16]. We have observed that euglycemia in the presence of insulin deficiency increases autophagy in the liver relative to that in the presence of a normal insulin level [12]; therefore, we hypothesized that euglycemia differentially influences the activities of AMPK and mTOR according to the insulin level in the liver, where glucose uptake is not dependent upon insulin. In addition, though insulin levels did not differ significantly among the diabetic rats, the extent of liver autophagy and the activity of Akt (a major insulin signaling molecule and an activator of mTOR) were significantly different between euglycemic-diabetic and hyperglycemic-diabetic rats [12]. Therefore, we also hypothesized that the glycemic level in the presence of insulin deficiency affects the molecules that transmit insulin signals, such as Akt and PTEN (a major negative regulator of the PI3 kinase/Akt signaling pathway, [17]), in the liver. Finally, because excessive autophagy induces apoptosis [18–20], we expected that increased apoptotic signaling may partially explain the significant loss of liver weight in euglycemic-diabetic rats. To test these hypotheses, molecular changes in the regulation of autophagy, insulin signaling, and apoptosis were studied in the livers of diabetic rats with different glycemic levels achieved by different diets, and these changes were compared to those of sham-operated control rats.

In the present study, we added an additional calorie-controlled group with restricted protein content (6 kcal% versus 19 kcal% in a standard chow) [12], to determine whether a protein restriction augments the effects of the calorie-controlled diet alone. In fact, protein restriction is often applied to patients with diabetes in clinical settings to prevent the progression of diabetic nephropathy [21–23]. Regarding the degree of protein restriction in the present study, we referred to other studies performed on protein restriction in diabetic rats [24, 25].

## 2. Materials and Methods

### 2.1. Animals and Study Design

**Figure 1:**
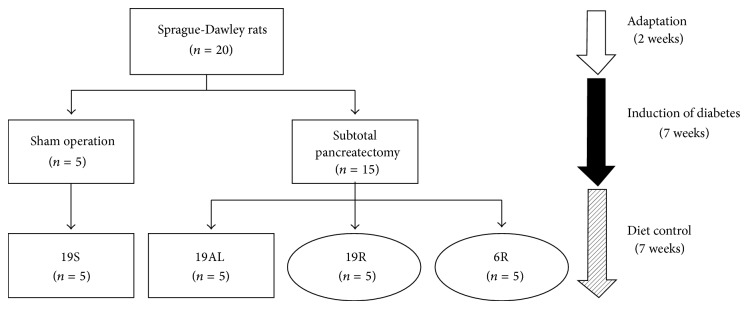
Animal study design. Square boxes:* ad libitum *diet; ovals: calorie-controlled diet; 19S: sham-operated rats fed standard chow* ad libitum* (protein 19.4, carbohydrate 68.8, and lipid 11.8 kcal%); 19AL: pancreatectomized diabetic rats fed standard chow* ad libitum*; 19R: pancreatectomized diabetic rats fed calorie-controlled standard chow to achieve euglycemia during the diet control period; 6R: pancreatectomized diabetic rats fed a calorie-controlled low protein diet (protein 6.0, carbohydrate 82.2, and lipid 11.8 kcal%) to achieve euglycemia during the diet control period.

Eleven-week-old, specific pathogen-free male Sprague-Dawley rats were purchased from Orient Bio (Sungnam, Korea). Upon arrival, they were weighed and housed individually for two weeks before surgery to allow adaptation. The rats were then divided into two groups and surgery was performed at 13 weeks of age as follows: five rats underwent a sham operation as the control group (19S), and 15 rats underwent subtotal pancreatectomy (Px group). After 7 weeks on an* ad libitum* diet to induce diabetes, the Px group was divided into thirds and fed the following diets for another 7 weeks:* ad libitum* group (19AL), calorie-control group (19R), and calorie and protein (calorie/protein) control group (6R). Throughout the study, all rats except the 6R group were fed a standard chow based on AIN-76A (Dyets Inc., Bethlehem, PA, USA; protein 19.4, carbohydrate 68.8, and lipid 11.8 kcal%). The 6R group was fed a low protein chow (modified AIN-76A, Dyets Inc.; protein 6.0, carbohydrate 82.2, and lipid 11.8 kcal%). The entire animal study design is demonstrated in Figure 1.

All rats were housed individually throughout the experiment and their daily food intake was measured. The 19S and 19AL groups were fed* ad libitum* during the entire study. The 19R and 6R groups were pair-fed (the same g per kg of body weight per day) with the 19S group. All rats were housed under a 12 h light-dark cycle (light on 08:00–20:00 h), at 20–23°C with a relative humidity of 40–65%. The rats had free access to tap water throughout the study. On the last day of the study after overnight fasting, rats were anesthetized by CO_2_, weighed immediately, and humanely euthanized. All animal protocols were approved by the Institutional Animal Care and Use Committee at Konkuk University.

### 2.2. Subtotal Pancreatectomy

To generate an insulin-deficient model of diabetes in adult rats, a subtotal pancreatectomy was performed at 13 weeks of age as described previously [12]. Briefly, the abdominal wall was opened under anesthesia using 0.7 mg/kg of Zoletil 50 (Virbac, Carros, France) and 0.2 mg/kg of Rompun (Bayer Korea, Ansan, Korea). Pancreatic tissue was removed carefully with a cotton tipped applicator from the spleen to 1 mm from the common bile duct without vascular injury. After surgery, the rats were placed in beds, covered, and exposed to infrared light to maintain normal body temperature. Control rats underwent a sham operation without the removal of pancreatic tissue.

### 2.3. Measurements of Food Intake, Body Weight, and Fasting Blood Glucose (FBG)

Food intake was individually measured daily and an average daily food intake (g per day) was calculated weekly. The daily food intake per kg of body weight of each rat was calculated using the average daily food intake and body weight from the previous week. Body weights were measured every weekend and on the last day of the study just before rats were euthanized. FBG levels (mg/dL) were measured in tail vein blood at 9 am every other week after overnight fasting using a portable glucometer (Caresens II, Gentrol Co., Incheon, Korea).

### 2.4. Measurements of Plasma Insulin and C-Peptide Levels

Immediately after CO_2_ anesthesia, blood samples were taken from the inferior vena cava. Plasma insulin and C-peptide levels were determined using radioimmunoassay kits (Millipore, Billerica, MA, USA) according to the manufacturer's instructions. Radioactivity was measured by using a *γ* counter (Beckman Coulter, Brea, CA, USA).

### 2.5. Measurement of Liver Weight and Preparation of Liver Tissue

After collecting blood samples, animals were euthanized and the liver was excised from the abdominal cavity, weighed, and immediately frozen in liquid nitrogen. Frozen liver tissues were ground to powder in liquid nitrogen in a mortar and stored at −80°C until use.

### 2.6. Western Blot Analysis

The frozen powdered liver tissue samples were homogenized in an ice-cold buffer containing 25 mM HEPES, 25 mM benzamidine, 100 mM sodium fluoride, 10 mM sodium pyrophosphate, 2 mM sodium orthovanadate, 1% triton X-100, 4 mM EDTA, 5 *μ*L/mL of phosphatase inhibitor, and 5 *μ*L/mL of protease inhibitor, sonicated for 1 minute, and centrifuged at 14,000 ×g, for 30 minutes at 4°C. Total protein concentrations were quantified using a BCA kit (Pierce, Rockford, IL, USA). The extracted proteins were separated on 8–13.5% SDS polyacrylamide gels and transferred to nitrocellulose membranes. Primary antibodies against total and phosphorylated AMPK (Cell Signaling Technology, CST, Denver, MA, USA; 1 : 5000), total and phosphorylated mTOR (CST; 1 : 1000), LC3B (CST; 1 : 1000), caspase-3 (CST; 1 : 1000), total and phosphorylated ERK-1 (CST; 1 : 5000), total and phosphorylated Akt (CST; 1 : 5000), and total and phosphorylated PTEN (CST; 1 : 1000) were applied overnight at 4°C. The membranes were then developed using horseradish peroxidase-conjugated anti-rabbit IgG (CST; 1 : 5000) followed by detection with ECL reagent (GE healthcare, Wauwatosa, WI, USA). The immunoreactive protein bands were quantified using Multi Gauge software version 3.1 (Fujifilm, Tokyo, Japan).

### 2.7. Statistical Analysis

Data are presented as means ± SD. Statistical analysis was performed using SPSS 18.0 software (SPSS Inc., Chicago, IL, USA). Statistical significance was evaluated using one-way ANOVA with Tukey's* post hoc* test, or an unpaired* t*-test. *P* values of less than 0.05 were considered statistically significant.

## 3. Results

### 3.1. Daily Food Intake, Body Weights, and Fasting Blood Glucose Levels

**Figure 2:**
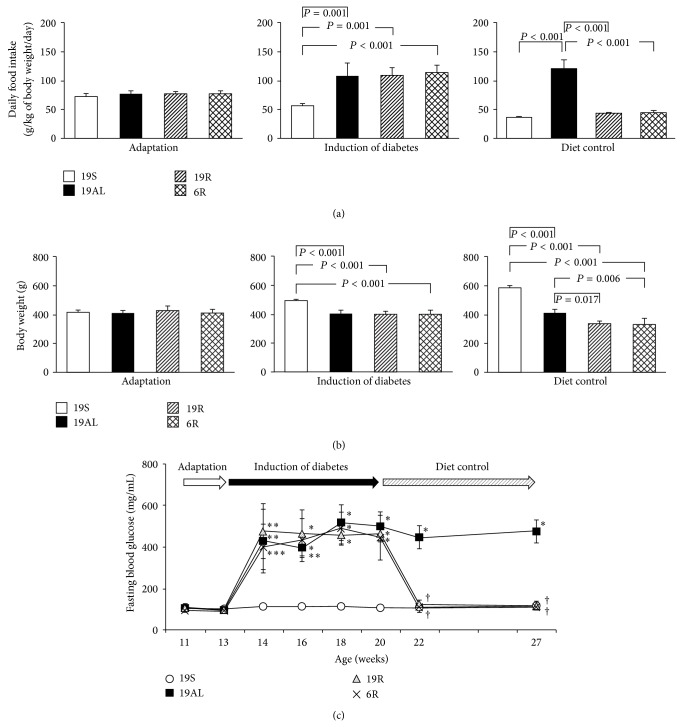
Changes in daily food intake (g per kg body weight per day), body weights, and fasting blood glucose (FBG) levels. (a) Mean daily food intake and (b) mean body weights during adaptation, induction of diabetes, and diet control periods. (c) Sequential changes in FBG levels throughout the study. Data are presented as means ± SD and were analyzed with one-way ANOVA followed by Tukey's* post hoc* test. 19S: sham-operated rats fed standard chow* ad libitum* (protein 19.4, carbohydrate 68.8, and lipid 11.8 kcal%); 19AL: pancreatectomized diabetic rats fed standard chow* ad libitum*; 19R: pancreatectomized diabetic rats fed calorie-controlled standard chow to achieve euglycemia during the diet control period; 6R: pancreatectomized diabetic rats fed a calorie-controlled low protein diet (protein 6.0, carbohydrate 82.2, and lipid 11.8 kcal%) to achieve euglycemia during the diet control period. ^∗^ *P* < 0.001, ^∗∗^ *P* < 0.01, and ^∗∗∗^ *P* < 0.05 versus 19S; ^†^ *P* < 0.001 versus 19AL.

To achieve euglycemia by diet control, pancreatectomized diabetic rats were fed as described in Section 2. The mean daily food intake per kg of body weight of all groups did not differ significantly during the adaptation period (Figure 2(a)). However, food intake by the Px groups increased by more than 2-fold during the period of diabetes induction as compared to the 19S group (*P* < 0.001). During the diet control period, food intake by the 19AL group was unchanged from the period of diabetes induction, while intake by the 19R and 6R groups decreased to that of the 19S group due to the controlled diet (Figure 2(a)). The 19AL group ate about three times more per body weight than the other groups during the diet control period (*P* < 0.001).

After the induction period while the mean body weights of all Px groups were unchanged, the mean body weights of the experimental rats changed as a result of the differences in diets and food intakes (Figure 2(b)). Compared to the 19S group, the mean body weights of the Px groups decreased significantly by about 20% during the period of diabetes induction (*P* < 0.001). The mean body weights of the 19R and 6R groups (338 ± 16 g and 330 ± 43 g, resp.) decreased further during the diet control period as compared to the 19S group (42 and 44% for the 19R and 6R groups, resp.; *P* < 0.001) and the 19AL group (18 and 19% for the 19R (*P* = 0.017) and 6R (*P* = 0.006) groups, resp.). The mean body weight of rats in the 19AL group (410 ± 26 g) was unchanged during the diet control period compared to the induction period but was decreased by 30% as compared to the 19S group (588 ± 12 g, *P* < 0.001) during the diet control period.

Figure 2(c) shows sequential changes in the FBG level of all groups throughout the entire study period and confirmed that both the calorie- and calorie/protein-controlled diets achieved euglycemia in the R groups. Before surgery, all groups were euglycemic (mean FBG levels were 19S: 104 ± 20; 19AL: 104 ± 18; 19R: 106 ± 11; 6R: 93.0 ± 10.9 mg/dL; *P* = 0.586). The FBG levels of the Px groups increased after surgery and remained elevated throughout the period of diabetes induction (19AL: 459 ± 52; 19R: 464 ± 68; 6R: 441 ± 41 mg/dL; *P* < 0.001 versus 19S at the last week of the diabetes induction period). However, the FBG levels of the 19R and 6R groups improved becoming euglycemic (113 ± 3.7 and 109 ± 10 mg/dL, resp.) like the 19S group (115 ± 22 mg/dL; *P* = 1 versus 19S) during the diet control period. In contrast, the 19AL group continued to be hyperglycemic (474 ± 55 mg/dL; *P* < 0.001 versus 19S, 19R, and 6R groups).

### 3.2. Plasma Insulin and C-Peptide Levels

**Figure 3:**
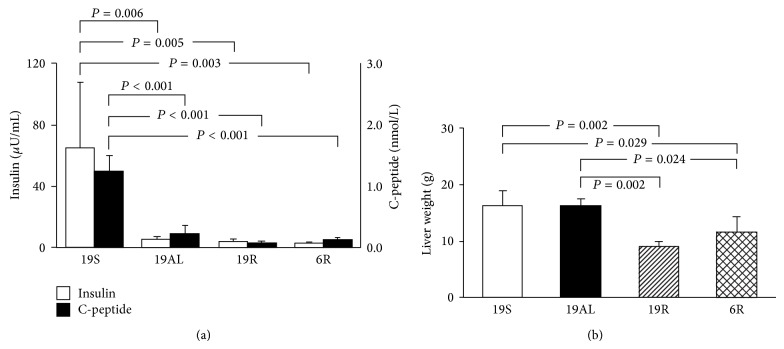
Endogenous insulin levels and liver weights at the end of the experiment. (a) Plasma insulin and C-peptide levels. (b) Liver weights. Data are presented as means ± SD and were analyzed with one-way ANOVA followed by Tukey's* post hoc* test. 19S: sham-operated rats fed standard chow* ad libitum* (protein 19.4, carbohydrate 68.8, and lipid 11.8 kcal%); 19AL: pancreatectomized diabetic rats fed standard chow* ad libitum*; 19R: pancreatectomized diabetic rats fed calorie-controlled standard chow to achieve euglycemia during the diet control period; 6R: pancreatectomized diabetic rats fed a calorie-controlled low protein diet (protein 6.0, carbohydrate 82.2, and lipid 11.8 kcal%) to achieve euglycemia during the diet control period.

To confirm that pancreatectomy generated insulin-deficient diabetic rats, plasma insulin and C-peptide levels were measured at the end of study. The mean plasma insulin levels of pancreatectomized rats were 8.9 (19AL), 6.5 (19R), and 5.5% (6R) that of the 19S group (*P* < 0.01) (Figure 3(a)). The mean plasma C-peptide levels of pancreatectomized rats were 18.2 (19AL), 6.7 (19R), and 10.4% (6R) that of the 19S group (*P* < 0.001) (Figure 3(a)). Plasma insulin and C-peptide levels among the Px groups did not differ significantly.

### 3.3. Liver Weights

Figure 3(b) shows that the mean liver weights of the 19R (9.0 ± 0.9 g) and 6R (11.5 ± 2.9 g) groups decreased significantly as compared to the 19S (16.3 ± 2.8 g) (45 (*P* = 0.002) and 29% (*P* = 0.029), resp.) or 19AL (16.4 ± 1.0 g) groups (45 (*P* = 0.002) and 30% (*P* = 0.024), resp.). By contrast, the liver weight of the 19AL group did not differ significantly from the 19S group.

### 3.4. Molecular Changes in the Regulation of Autophagy in Liver Tissue

**Figure 4:**
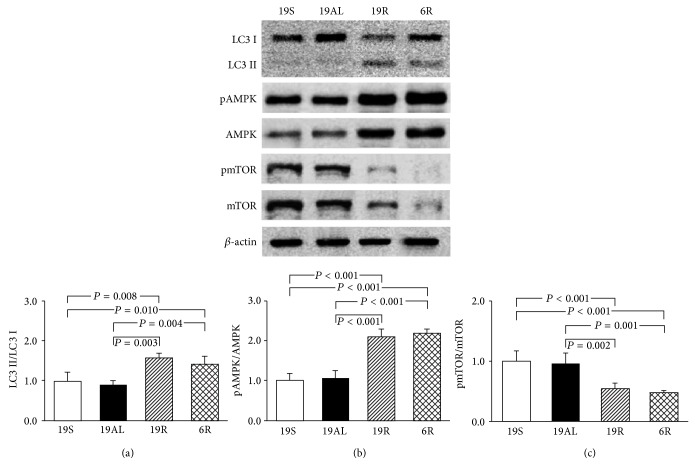
Western blot analyses related to the regulation of autophagy in liver tissue. (a) The ratio of LC3 II to LC3 I. (b) The ratio of pAMPK to AMPK. (c) The ratio of pmTOR to mTOR. The blots shown are representative of triplicates. Data are presented as means ± SD and were analyzed with one-way ANOVA followed by Tukey's* post hoc* test. 19S: sham-operated rats fed standard chow* ad libitum* (protein 19.4, carbohydrate 68.8, and lipid 11.8 kcal%); 19AL: pancreatectomized diabetic rats fed standard chow* ad libitum*; 19R: pancreatectomized diabetic rats fed calorie-controlled standard chow to achieve euglycemia during the diet control period; 6R: pancreatectomized diabetic rats fed a calorie-controlled low protein diet (protein 6.0, carbohydrate 82.2, and lipid 11.8 kcal%) to achieve euglycemia during the diet control period.

The ratio of LC3 II to I, an index of autophagy in liver tissue, is shown in Figure 4(a). The ratio of the 19R and 6R groups increased significantly as compared to the 19S (1.6- (*P* = 0.008) and 1.4-fold (*P* = 0.010), resp.) and 19AL groups (1.7- (*P* = 0.003) and 1.6-fold (*P* = 0.004), resp.). The two R groups did not differ significantly (Figure 4(a)).

The phosphorylation levels of AMPK and mTOR were examined to assess changes in factors that regulate autophagy. The phosphorylation of AMPK in the 19R and 6R groups increased significantly as compared to the 19S (2.1- and 2.2-fold, resp.; *P* < 0.001) and 19AL groups (2.0- and 2.1-fold, resp.; *P* < 0.001). The two R groups did not differ significantly (Figure 4(b)). Phosphorylation of mTOR in the 19R and 6R groups decreased significantly as compared to the 19S (46 and 52%, resp.; *P* < 0.001) and 19AL groups (44 (*P* = 0.001) and 50% (*P* = 0.002), resp.). The two R groups did not differ significantly (Figure 4(c)). The ratio of LC3 II to I and the levels of AMPK and mTOR phosphorylation did not differ significantly between the 19S and 19AL groups (Figures 4(a)–4(c)).

### 3.5. Comparison of Insulin Signaling in Liver Tissue

**Figure 5:**
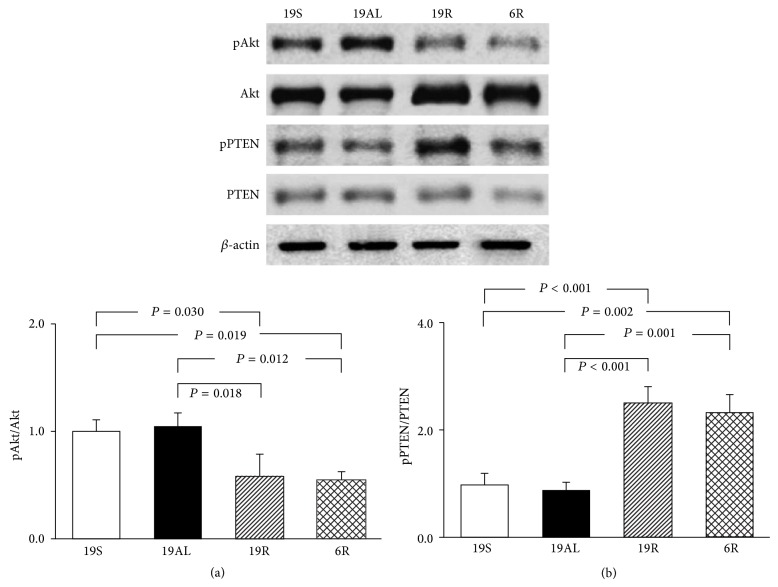
Western blot analyses related to the insulin signaling in liver tissue. (a) The ratio of pAkt to Akt. (b) The ratio of pPTEN to PTEN. The blots shown are representative of triplicates. Data are presented as means ± SD and were analyzed with one-way ANOVA followed by Tukey's* post hoc* test. 19S: sham-operated rats fed standard chow* ad libitum* (protein 19.4, carbohydrate 68.8, and lipid 11.8 kcal%); 19AL: pancreatectomized diabetic rats fed standard chow* ad libitum*; 19R: pancreatectomized diabetic rats fed calorie-controlled standard chow to achieve euglycemia during the diet control period; 6R: pancreatectomized diabetic rats fed a calorie-controlled low protein diet (protein 6.0, carbohydrate 82.2, and lipid 11.8 kcal%) to achieve euglycemia during the diet control period.

The phosphorylation levels of Akt and PTEN were determined to assess the insulin signaling level, which can modulate the extent of autophagy (Figure 5). The phosphorylation of Akt decreased significantly in the 19R and 6R groups as compared to the 19S (42 (*P* = 0.030) and 46% (*P* = 0.019), resp.) and 19AL groups (44 (*P* = 0.012) and 48% (*P* = 0.018), resp.). The two R groups did not differ significantly (Figure 5(a)). The phosphorylation of PTEN increased significantly in the 19R and 6R groups as compared to the 19S (2.5- (*P* < 0.001) and 2.3-fold (*P* = 0.002), resp.) and 19AL groups (2.9- and 2.7-fold, resp.; *P* < 0.001). The two R groups did not differ significantly (Figure 5(b)), and the phosphorylation of Akt and PTEN did not differ significantly between the 19S and 19AL groups (Figures 5(a) and 5(b)).

### 3.6. Molecular Changes in the Regulation of Cell Growth/Proliferation and Apoptosis in Liver Tissue

**Figure 6:**
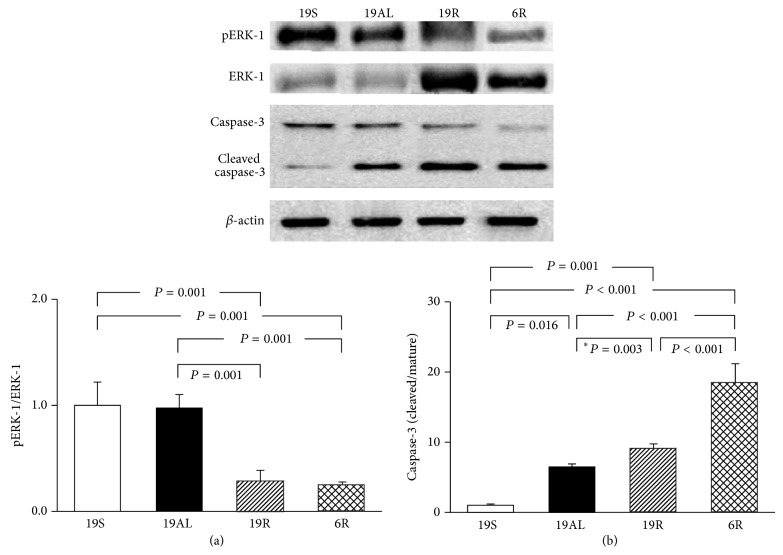
Western blot analyses related to the regulation of cell growth/proliferation and apoptosis in liver tissue. (a) The ratio of pERK-1 to ERK-1. (b) The ratio of cleaved to mature caspase-3. The blots shown are representative of triplicates. Data are presented as means ± SD and were analyzed with one-way ANOVA followed by Tukey's* post hoc* test unless marked with an asterisk indicating that the unpaired* t*-test was used. 19S: sham-operated rats fed standard chow* ad libitum* (protein 19.4, carbohydrate 68.8, and lipid 11.8 kcal%); 19AL: pancreatectomized diabetic rats fed standard chow* ad libitum*; 19R: pancreatectomized diabetic rats fed calorie-controlled standard chow to achieve euglycemia during the diet control period; 6R: pancreatectomized diabetic rats fed a calorie-controlled low protein diet (protein 6.0, carbohydrate 82.2, and lipid 11.8 kcal%) to achieve euglycemia during the diet control period.

The phosphorylation of ERK-1 and the cleavage ratio of caspase-3 (the ratio of cleaved to uncleaved caspase-3) were measured to assess changes in factors affecting the regulation of cell growth/proliferation and apoptosis, respectively (Figure 6). The phosphorylation of ERK-1 in the 19R and 6R groups decreased significantly as compared to the 19S (72 and 75%, resp.; *P* = 0.001) and 19AL groups (71 and 75%, resp.; *P* = 0.001) (Figure 6(a)). The two R groups did not differ significantly, and the phosphorylation of ERK-1 in the 19AL group did not differ significantly from the 19S group.

The cleavage ratio of caspase-3 increased in all Px groups compared to the 19S group: 19AL: 6.4-fold, *P* = 0.016; 19R: 9.1-fold, *P* = 0.001; and 6R: 18.3-fold, *P* < 0.001 (Figure 6(b)). There were also significant differences in the cleavage ratio between the Px groups: the ratio of the 19R and 6R groups increased significantly by 1.4- (*P* = 0.003) and 2.9-fold (*P* < 0.001), respectively, compared to the 19AL group. The ratio of the 6R group was highest among all the Px groups (2-fold higher than the 19R group, *P* < 0.001).

## 4. Discussion

Achieving euglycemia in diabetic patients has been the ultimate goal of treatment. However, the increased mortality associated with the intensive glycemic control required to achieve euglycemia has emerged as a new concern whose molecular mechanisms, and the organs involved, are still largely unknown.

In the present study, we demonstrated that achieving euglycemia in insulin deficiency (both the 19R and 6R groups) was accompanied by a loss of a critical amount of functional mass of the liver via increased autophagy, as compared with euglycemia in the presence of normal physiologic levels of insulin (19S group). Autophagy was increased in both R groups through increased AMPK and decreased mTOR (via increased PTEN and decreased Akt activation) activation. There was also an increase in the amount of cleaved (activated) caspase-3, an executor of apoptosis (Figures 4–6).

Autophagy plays a major role in cell survival under stress conditions. However, based on recent studies, as well as the data presented here, autophagy can kill a cell if essential components are rapidly consumed [26, 27]. Autophagy is regulated by nutrient levels in a cell that affect the activities of AMPK and mTOR, the two major regulators of the autophagic process [15]. However, our results showed that the activation level of AMPK in hepatocytes differed significantly under identical euglycemic conditions (both R groups versus the 19S group). This suggests that the energy level in hepatocytes may be affected by insulin as shown in skeletal muscle [28], despite glucose uptake being independent of insulin in liver tissue but not in skeletal muscle tissue. Insulin promotes glycolysis and ATP production in hepatocytes. This may explain why the activation of AMPK in both insulin-deficient R groups is greater than that of the 19S group under the same euglycemic conditions. Another possibility for activating AMPK in insulin-deficient hepatocytes is by 3-phosphoglycerate (3-PG), a glycolysis intermediate, via AMPKK [29]. Insulin regulates the flux of glycolysis and AMPK inhibits gluconeogenesis. Thus, we speculate that 3-PG may accumulate because the flux of the glycolytic pathway is retarded due to insulin deficiency and because the flux of the gluconeogenic pathway is inhibited by activated AMPK. Taken together, euglycemia in the presence of insulin deficiency induces a starvation-like effect in the liver compared to that with physiologic insulin levels. This is shown by the activation of AMPK and subsequent excessive autophagy in both R groups.

The lower mTOR activation in the 19R group than in the 19S group, even on the isocaloric and isoprotein diets, may be the result of insulin deficiency that decreased Akt (an activator of mTOR) [30] phosphorylation and activated PTEN (a major negative regulator of the PI3 kinase/Akt signaling pathway, [17]) in the 19R group compared to the 19S group. Furthermore, AMPK is activated by energy depletion and suppresses mTOR activity thus decreasing the inhibitory influence of mTOR over autophagy [31, 32].

PTEN phosphorylation increases the activity and stability of PTEN itself; however, the total PTEN levels did not differ among the experimental groups. Considering that PTEN expression is upregulated in podocytes under hyperglycemic conditions (30 mmol/L) relative to under normoglycemic conditions (5.6 mmol/L) [33], we speculated that PTEN in both R groups could be maintained at a similar level to those of the other two groups due to the increased stability, though the expression of PTEN itself might be downregulated in euglycemic conditions.

Because autophagy is associated with the activity of ERK, a major signaling molecule for cell growth/proliferation through the inhibition of transcription factor EB (TFEB, a master regulator of lysosomal biogenesis and autophagy, [34, 35]), we investigated ERK-1 activity (Figure 6(a)). As expected, ERK-1 activity decreased in both R groups compared to the 19S group. This finding is consistent with the increased autophagy in both R groups.

Interestingly, while starvation-induced autophagic death, but not apoptosis, was observed in the liver of patients with anorexia nervosa that are severely undernourished [36, 37], both euglycemic R groups, in a comparable state of starvation because of insulin deficiency, showed increased apoptotic signaling as well as excessive autophagy (Figures 4(a) and 6(b)). This suggests that mitochondrial damage and the release of cytochrome *c* may occur in hepatocytes under euglycemic insulin deficiency. This, in turn, would lead to caspase-3 activation, which was not observed in anorexia nervosa without diabetes [36, 37]. Mitochondrial performance and mass were markedly reduced in the soleus muscle of STZ-induced diabetic mice, but not in high fat diet induced diabetic mice with hyperinsulinemia [28], indicating the significance of insulin in maintaining mitochondrial function. Based on these findings, we propose that, while euglycemia can be achieved without resolution of insulin deficiency, which is common even in patients with T2D, this is not beneficial to diabetic patients in terms of the survival of hepatocytes and the maintenance of the functional mass of the liver.

In this study, we investigated the molecular mechanisms by which hyperglycemia in insulin deficiency (19AL group) protected the liver against critical weight loss compared to euglycemia in insulin deficiency (both R groups). Hyperglycemia due to the* ad libitum* diet in the 19AL group resulted in an autophagy level similar to that of the 19S group through decreased AMPK and increased mTOR phosphorylation (Figure 4). The cleavage of caspase-3 decreased as compared to both R groups (Figure 6) even though all Px groups did not differ significantly in insulin levels (Figure 3(a)).

Hyperphagia has been considered to be a pathologic eating habit in diabetic patients. Because hyperphagia results in hyperglycemia in the presence of insulin deficiency, which is associated with increased microvascular complications, diet control has been a major strategy for diabetes care [8, 38, 39]. In fact, hyperglycemia is harmful to endothelial cells because of glucotoxicity. Endothelial cells use GLUT-1 for glucose uptake from the blood [40, 41]. The activity of GLUT-1 is not dependent on insulin but rather on the level of glycemia. Therefore, as the glycemic level increases, endothelial cells become more exposed to glucotoxicity, and microvascular complications are more prone to develop. Livers from rats in the hyperglycemic 19AL group were protected against the loss of functional mass through decreased AMPK and increased mTOR and Akt (a major insulin receptor signaling molecule, [42]) phosphorylation, as compared to both R groups, in spite of insulin deficiency. This implies that hyperglycemia, which is harmful to diabetic patients, activates Akt and inhibits autophagy and apoptosis like insulin [43, 44], but this effect is independent of the insulin level [45]. Therefore, we speculate that hyperphagia-induced hyperglycemia in the presence of insulin deficiency may not always be deleterious but rather has the beneficial effect of limiting autophagic and apoptotic cell death in the liver, but at the cost of increasing microvascular complications. Our data may explain why the conventional care group (mean HbA1c, 7.5%) showed a lower overall mortality rate than the intensive glycemic control group (mean HbA1c, 6.4%) in the Action to Control Cardiovascular Risk study [46].

Finally, we investigated the difference in molecular changes between the two control diets (normal or reduced protein content to achieve euglycemia). Diabetic patients are easily undernourished, especially for protein, because of the strict diet control needed to control hyperglycemia and to prevent the progression of diabetic nephropathy [21–23]. The only difference found between the two R groups was in the level of cleaved caspase-3 that was significantly higher in the 6R than in the 19R group. We speculate that, despite euglycemia in the presence of insulin deficiency, the activation of apoptotic signals may be stronger in the liver when fed a protein restricted diet than when fed a non-protein restricted diet. This finding warrants further study to determine the underlying mechanism.

**Figure 7:**
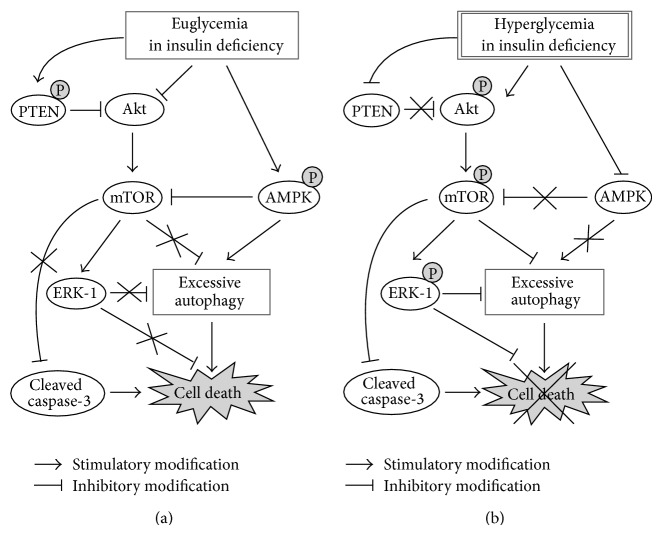
Proposed molecular mechanisms for (a) the loss or (b) the preservation of liver weight by glycemic levels in the presence of insulin deficiency. In the liver of euglycemic rats with insulin deficiency, AMPK is activated and the activity of Akt, a major insulin signaling molecule, decreases (the decreased Akt activity is also influenced by PTEN activation), compared with those in hyperglycemic rats with insulin deficiency. The increased AMPK and decreased Akt activities, in turn, decrease the activity of mTOR. As a result, excessive autophagy is induced not only by the activated AMPK but also by the inhibition of protective role of mTOR against autophagy through ERK-1 activation and caspase-3 inhibition. Then excessive autophagy leads to hepatic cell death, partially via apoptosis mediated by caspase-3 activation, resulting in the loss of liver weight in euglycemic rats with insulin deficiency. On the other hand, in the liver of hyperglycemic rats with insulin deficiency, AMPK is not activated while Akt and mTOR are activated, compared with those in euglycemic rats with insulin deficiency. Thus, excessive autophagy and hepatic cell death are prevented not only because of the protective role of mTOR against autophagy but also because of the low AMPK activity, resulting in the preservation of liver weight in hyperglycemic rats with insulin deficiency.

Taken together, euglycemia achieved by feeding a calorie- or calorie/protein-controlled diet in insulin deficiency can be detrimental to the survival of hepatocytes due to energy depletion and low insulin receptor signaling in the liver parenchyma, even when the calories ingested by the diabetic rats were the same as the daily intake per body weight of sham-operated controls. In contrast, the hyperglycemia from feeding an* ad libitum* diet in the presence of insulin deficiency retained liver weight due to an insulin receptor signaling level that was comparable to sham-control rats. We simplified these proposed molecular mechanisms as Figure 7.

Overall, we have demonstrated the molecular mechanisms by which euglycemia differentially affects the liver, depending on the insulin status. We have also shown that hyperglycemia has a protective effect on the liver in the presence of insulin deficiency. Therefore, we propose that achieving euglycemia by any kinds of intervention without resolution of insulin deficiency should be avoided. It is important for care-givers of diabetic patients to understand that the hyperphagia seen in the presence of insulin deficiency is to compensate for the insulin deficiency in the liver, indicating that the present paradigm for diet control in diabetes care should be reevaluated.
